# Analysis of Nonunion in Conservatively Managed Anterior Tear Drop Fractures of C2 Vertebra

**DOI:** 10.3390/jcm10092037

**Published:** 2021-05-10

**Authors:** Sung-Kyu Kim, John M. Rhee, Eric T. Park, Hyoung-Yeon Seo

**Affiliations:** 1 Department of Orthopaedic Surgery, Chonnam National University Medical School and Hospital, Gwangju 61469, Korea; 1976skkim@daum.net; 2 Emory Spine Center, Department of Orthopaedic Surgery, Emory University, Atlanta, GA 30303, USA; jmrhee@emory.edu; 3 Department of Biology, College of Arts and Sciences, Emory University, Atlanta, GA 30322, USA; eric.park@alumni.emory.edu

**Keywords:** tear drop fracture, axis, conservative treatment, nonunion, surgical treatment

## Abstract

Many anterior C2 (2nd cervical vertebra) tear drop (TD) fractures can be successfully managed with conservative treatment. However, due to the occurrence of nonunion, large-sized or complex anterior C2 TD fractures undergo surgical treatment. To date, no surgical treatment guidelines are available about anterior C2 TD fractures. Therefore, we performed this study to investigate the factors that may affect nonunion for anterior C2 TD fractures and to suggest surgical treatment guidelines. Thirty-three patients with anterior C2 TD fractures, who underwent conservative treatment and had a minimum 1-year follow-up, were divided into union (*N* = 26) and nonunion (*N* = 7) groups. Their radiological and clinical data were analyzed retrospectively and compared between the two groups. The avulsion fracture ratio (29.5% vs. 43.3%, *p* < 0.05) and fracture displacement (3.6 mm vs. 5.1 mm, *p* < 0.05) were higher in the nonunion group compared to the union group. Incidence of associated C2 injury was higher in the nonunion group compared to the union group (15.4% vs. 57.1%, *p* < 0.05). Union status was negatively correlated with associated C2 injury (correlation coefficient, CC = −0.398, *p* < 0.05). Our results suggest that surgical treatment could be considered for anterior C2 TD fractures with an avulsion fracture ratio > 43%, fracture displacement > 5 mm, or associated C2 injury.

## 1. Introduction

Tear drop (TD) fractures of the cervical spine are divided into flexion TD fractures caused by flexion-compression force and extension TD fractures (avulsion fracture) caused by hyperextension. Flexion TD fractures commonly occur at the C4–C7 (cervical) vertebra. In comparison, extension TD fractures occur more commonly at C2 or C3. Anterior TD fractures of the C2 vertebra are a relatively rare cervical spine injury. Its incidence is about 9–12% of upper cervical spine injuries and 1–3% of all cervical spine injuries [1,2,3,4,5,6]. Anterior C2 TD fractures are commonly caused by extension injury that differs in several ways from TD fractures in the lower cervical spine. To date, a few studies, including series with a small number of cases or case reports, have reported treatment methods and outcomes for anterior C2 TD fractures [1,2,3,4]. Most anterior C2 TD fractures can be successfully managed with conservative treatment because an extension TD fracture is more stable [7,8]. However, a close analysis of previous studies showed that anterior C2 TD fractures successfully managed with conservative treatment were small-sized or simple anterior TD fractures. Furthermore, a few case reports have suggested that if anterior C2 TD fractures are huge or massive, it is better to reconstruct the anterior column through surgery [9,10,11,12,13,14]. However, the exact criteria for determining indications for surgical treatment of large-sized anterior C2 TD fractures have not been provided and are controversial.

It has been our experience that the larger the fracture size is, or the greater the fracture displacement is, the more likely nonunion is to occur. To the best of our knowledge, however, no one has previously investigated the relationship between the fracture characteristics of anterior C2 TD fractures and nonunion outcomes. Moreover, there have been no studies examining factors that influence nonunion for conservatively managed anterior C2 TD fractures. Therefore, we performed the current study to suggest guidelines for determining surgical treatment of anterior C2 TD fractures.

## 2. Materials and Methods

A total of 60 patients with anterior or posterior TD fractures of the C2 body were identified from the databases of 4 national trauma centers housed at tertiary university hospitals between the years 2000 and 2017. The inclusion criteria for this study were as follows: acute trauma history, anterior C2 TD fracture as diagnosed using lateral X-ray and sagittal 2-dimensional (2-D) reconstructed computed tomography (CT) scan, conservative treatment, and a minimum 1-year follow-up. Among them, 33 patients met the inclusion criteria and were included in this study.

All 33 patients with anterior C2 TD fractures were divided into union or nonunion groups depending on fusion status at the 1-year follow-up point. Fusion status was evaluated by neutral, flexion, and extension lateral radiographs. The criteria for fusion were as follows: formation of a bony bridge and no motion between the anterior C2 TD fracture and the anteroinferior portion of the C2 body. Plain radiographs, 2-D reconstructed CT, magnetic resonance imaging (MRI), and medical records were analyzed retrospectively and the results between the two groups were compared.

**Figure 1:**
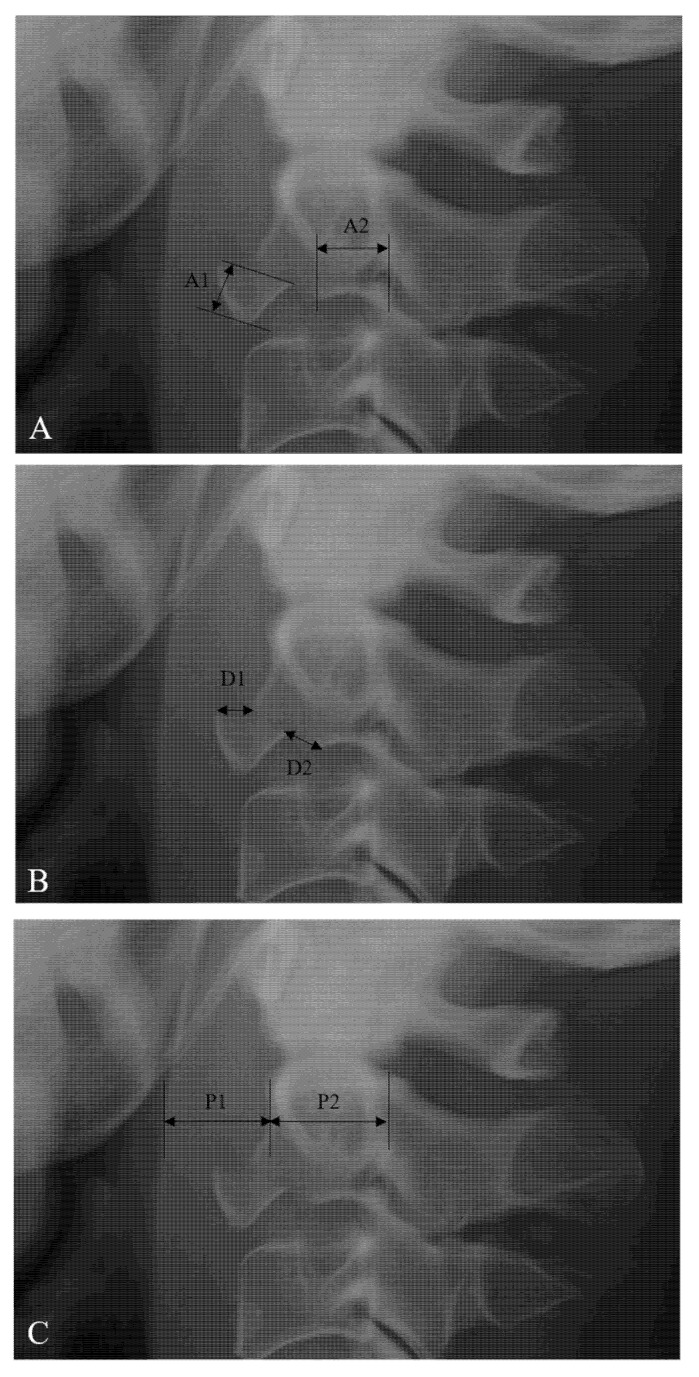
(**A**) The method of measuring the avulsion fracture ratio of anterior C2 (2nd cervical vertebra) tear drop (TD) fractures. The sagittal diameter of the inferior C2 endplate includes A1 and A2 (inferior endplate diameter of C2 TD fragment (A1) and remaining C2 body (A2)). Avulsion ratio of anterior C2 TD fracture = A1/(A1 + A2) × 100%. (**B**) The method of measuring the fracture displacement of anterior C2 TD fractures. The displacement of the fragment is measured at the superior point (D1) and the posteroinferior point (D2). Fracture displacement of anterior C2 TD fracture = (D1 + D2)/2. (**C**) The method of measuring the prevertebral soft tissue thickness (PSVT). The sagittal diameter of PSVT (P1) and C2 body (P2) is measured at the C2 body midpoint. Ratio of PSVT at anterior C2 TD fracture = P1/P2.

The avulsion fracture ratio of anterior C2 TD fractures was measured from lateral X-ray images (Figure 1A) [9]. The sagittal diameter of the inferior C2 endplate includes A1 and A2 (inferior endplate diameter of C2 TD fragment (A1) and remaining C2 body (A2)). The avulsion fracture ratio of anterior C2 TD fractures was defined as A1/(A1 + A2) × 100%. The fracture displacement of anterior C2 TD fractures was measured from lateral X-ray (Figure 1B) [7,8,9]. The displacement of the anterior C2 TD fragment was measured at the superior point (D1) and the posteroinferior point (D2). The fracture displacement of anterior C2 TD fractures was defined as (D1 + D2)/2. The prevertebral soft tissue thickness (PSVT) was measured from lateral X-ray (Figure 1C) [15]. The sagittal diameter of PSVT (P1) and the C2 body (P2) was measured at the C2 body midpoint. The ratio of PSVT was defined as P1/P2. The severity of PSVT at C2 was classified on a scale of grade 1 (mild, <0.4), grade 2 (moderate, 0.4–0.7), and grade 3 (severe, >0.7). The characteristics of the anterior C2 TD fractures, including associated C2 injury, associated C1 injury, associated C3–7 or thoracolumbar injuries, and neurologic status, were investigated. Clinical outcomes were evaluated using Odom’s criteria [16] and the visual analog scale (VAS) for neck pain [17]. All radiographic data were examined independently by two spine surgeons. Each independent observer checked the avulsion fracture ratio, fracture displacement, and severity of PSVT twice, and the average of the four measurements was used as the final result.

Statistical analysis was performed using the independent *t*-test, paired *t*-test, chi-squared test, and Pearson correlation test. A *p*-value of less than 0.05 was considered significant.

## 3. Results

### 3.1. Demographic Data

The mean age at the time of diagnosis was 51.6 years (range: 21–77 years). The mean follow-up period was 14.7 months (range: 12–43 months). Twenty-four patients were men and nine patients were women. Regarding the injury mechanism, 13 patients (39.4%) were involved in a traffic accident, 12 (36.4%) experienced a fall from height, and 8 (24.2%) slipped and fell. At admission, all 33 patients (100%) were neurologically intact. In terms of conservative treatment methods, 12 patients (36.4%) wore a Philadelphia brace, 14 patients (42.4%) wore a Miami brace, 5 patients (15.2%) wore a Minerva brace, and 2 patients (6.0%) wore a Halo vest.

**Figure 2:**
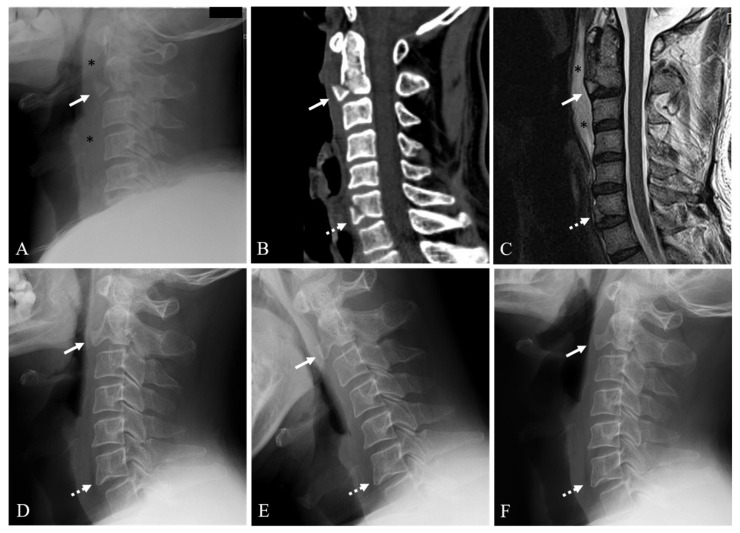
Lateral X-ray (**A**) and sagittal 2-dimensional (2-D) reconstructed computed tomography (CT) scan (**B**) showing anterior C2 tear drop (TD) fracture (white arrow), C6 fracture (dotted white arrow), and prevertebral soft tissue swelling (asterisks). Sagittal magnetic resonance imaging (**C**) showing anterior C2 TD fracture with 38% avulsion fracture ratio (white arrow), C6 fracture (dotted white arrow), and prevertebral hematoma (asterisks). At 12-month follow-up after Philadelphia brace, neutral (**D**), flexion (**E**), and extension (**F**) lateral X-rays showing solid fusion of anterior C2 TD fracture (white arrow) and C6 fracture (dotted white arrow).

**Figure 3:**
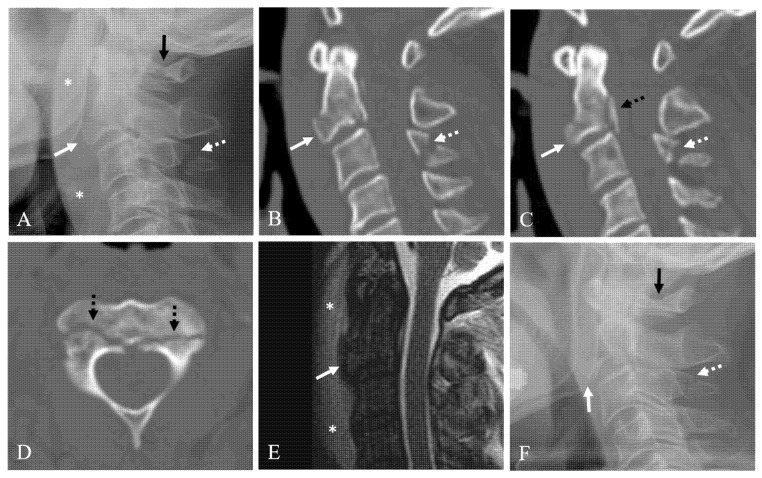
Lateral X-ray (**A**) and sagittal 2-dimensional (2-D) reconstructed computed tomography (CT) scans (**B**,**C**) showing anterior C2 tear drop (TD) fracture with 75% avulsion fracture ratio (white arrow), prevertebral soft tissue swelling (asterisks), C1 posterior arch fracture (dark arrow), posterior C2 body vertical fracture (dotted dark arrow), and C3 spinous process fracture (dotted white arrow). Axial CT scan (**D**) showing bilateral pedicle fracture (dotted dark arrows). Sagittal magnetic resonance imaging (**E**) showing anterior C2 TD fracture of C2 (white arrow) and prevertebral hematoma (asterisks). (**F**) At 12-month follow-up after Minerva brace, lateral X-ray showing displaced nonunion of anterior C2 TD fracture (white arrow) compressing the pharynx, and nonunion of C3 spinous process fracture (dotted white arrow) but solid fusion of C1 posterior arch fracture (dark arrow).

Twenty-six patients (78.8%) were in the union group (Figure 2) and seven patients (21.2%) were in the nonunion group (Figure 3). The demographic data and information for the union and nonunion groups of conservatively managed anterior C2 TD fractures are summarized in Table 1. Mean age at the time of diagnosis was not statistically different between the union and nonunion groups (51.5 years vs. 51.9 years, *p* = 0.961). Mean follow-up time was not statistically different between the union and nonunion groups (15.4 months vs. 12.0 months, *p* = 0.282). At the time of diagnosis, all patients were neurologically intact.

### 3.2. Radiological Outcomes

The correlation coefficients for intra-observer reliability for avulsion fracture ratio, fracture displacement, and PSVT severity were 0.721, 0.725, and 0.743 (all, *p* < 0.001), respectively, which indicated a strong reliability. The correlation coefficients for inter-observer reliability for avulsion fracture ratio, fracture displacement, and PSVT severity were 0.581, 0.592, and 0.597 (all, *p* < 0.001), respectively, which indicated a moderate reliability. The avulsion fracture ratio was significantly higher in the nonunion group compared to the union group (29.5% vs. 43.3%, *p* < 0.05). Fracture displacement was significantly higher in the nonunion group compared to the union group (3.6 mm vs. 5.1 mm, *p* < 0.05). In terms of PSVT severity, 8 patients were grade 1 (30.8%), 4 patients were grade 2 (15.4%), and 14 patients were grade 3 (53.8%) in the union group. In the nonunion group, 2 patients were grade 1 (28.6%), 1 patient was grade 2 (14.3%), and 4 patients were grade 3 (57.1%); therefore, the PSVT severity was not statistically different between the two groups (*p* = 0.988). The incidence of associated C1 injury (3.8% vs. 12.5%, *p* = 0.421) and associated C3–7 or thoracolumbar injury (26.9% vs. 28.6%, *p* = 1.000) was not statistically different between the union and nonunion groups. However, the incidence of associated C2 injury was significantly higher in the nonunion group compared to the union group (15.4% vs. 57.1%, *p* < 0.05). The Pearson correlation test results showed that the union status in conservatively managed anterior C2 TD fractures was negatively correlated with associated C2 injury (correlation coefficient, CC = −0.398, *p* < 0.05).

### 3.3. Clinical Outcomes

According to clinical outcomes using Odom’s criteria, all 26 union patients showed excellent (*N* = 16, 61.5%) and good (*N* = 10, 38.5%) results. However, four nonunion patients (57.1%) showed good results and three nonunion patients (42.9%) showed fair results. The difference in satisfactory outcome was statistically significant between the union and nonunion groups (100% vs. 57.1%, *p* < 0.001) (Table 2). The VAS score for neck pain was significantly improved in the union group (4.1 vs. 1.2, *p* < 0.001), but not in the nonunion group (4.3 vs. 3.6, *p* = 0.094) (Table 3). At the last follow-up, the VAS score for neck pain was significantly lower in the union group compared to the nonunion group (1.2 vs. 3.6, *p* < 0.01). In terms of dysphagia, one patient complained of difficulty with swallowing solid foods due to displaced nonunion compressing the pharynx. However, the patient refused surgery. The remaining two patients with fair results did not want additional treatment despite the discomfort.

## 4. Discussion

### 4.1. Analysis of Our Results

In this study, we retrospectively analyzed clinical and radiological data of 33 anterior C2 TD fractures in patients who underwent conservative treatment and had a minimum 1-year follow-up. All 33 patients were assigned to the union group or the nonunion group based on their C2 union status at the 1-year follow-up time point. We found that the nonunion group had a higher avulsion fracture ratio of 43.3% and a fracture displacement of 5.1 mm; these findings were statistically significant compared to the union group. The nonunion group had a significantly higher incidence of associated C2 injury. Union status in conservatively managed anterior C2 TD fractures was negatively correlated with associated C2 injury. Finally, the nonunion group showed less satisfactory clinical outcomes compared to the union group. Based on the current findings, we suggest that surgical treatment could be considered for anterior C2 TD fractures with an avulsion fracture ratio > 43%, fracture displacement > 5 mm, or presence of an associated C2 injury. To the best of our knowledge, this is the first study to suggest guidelines for determining surgical treatment of anterior C2 TD fractures.

### 4.2. Outcomes of Previous Studies

In general, C2 fractures heal well with conservative treatment. In a review of the literature, the success rate of conservative treatment for C2 fractures was 78.4% [18]. Like other C2 fractures, conservative treatment has been used as the standard treatment for anterior C2 TD fractures. Previous studies with mostly small case numbers and small-sized fractures have reported satisfactory outcomes for anterior C2 TD fractures managed conservatively [4,7,8]. Kim et al. reported four extension-type C2 tear drop fractures out of 25 cervical tear drop fractures. They reported that all C2 tear drop fractures were well treated with conservative treatment [19]. On the other hand, a few case reports have described surgical treatments for huge or large-sized anterior C2 TD fractures [9,10,11,12,13,14]. According to the review of the literature, as the fracture displacement of C2 increased, the proportion of nonunion after conservative treatment increased. In addition, when the fracture displacement was more than 5 mm, surgery was performed immediately [18]. However, their suggestions were based on the overall C2 fracture, including dens fracture, and the exact criteria for surgical treatment of C2 anterior tear drop fracture were not provided.

### 4.3. Comparison with Previous Studies

In this study, the nonunion group of conservatively managed anterior C2 TD fractures showed a significantly higher avulsion fracture ratio of 43.3% compared to 29.5% in the union group. According to a few case reports [9,10,11,12,13,14] and case series with a small case number [20,21], anterior TD fractures that involve about 50% of the inferior C2 endplate need to be treated surgically to avoid nonunion. Our result showed a 7% lower avulsion fracture ratio compared to that in previous studies. In addition, our results were obtained with a larger number of cases through a multicenter study compared to prior studies. We believe the current study includes the largest number of cases of anterior C2 TD fractures compared to papers published so far. We also showed that nonunion may occur if the fracture displacement is over 5.1 mm, which was not mentioned in previous studies. These two radiological findings might be considered to cause nonunion of conservatively managed anterior C2 TD fractures and may serve as guidelines for determining surgical treatment of anterior C2 TD fractures.

### 4.4. Injury Mechanism and Associated Injuries

Anterior C2 TD fractures can occur alone or in conjunction with other associated spine injuries including C2, C1, and C3–7 or thoracolumbar spine. Since these injuries may have an effect on treatment methods and outcomes, special attention is needed to assess these associated injuries. Anterior C2 TD fracture is reported to be caused by extension injury. If the extension injury is more severe, anterior C2 TD fracture occurs, followed by C2 body or posterior bony elements. Anterior TD fractures are much more unstable if accompanied by fractures of the C2 body or posterior elements. If conservative treatment is performed in such cases, the risk of treatment failure is high and may lead to a displaced nonunion, such as that in Figure 3. In our study, the nonunion group had a significantly higher incidence rate of associated C2 injury of 57.1% compared to 15.4% of the union group. In addition, the Pearson correlation test showed that union status was negatively correlated with associated C2 injury (CC = −0.398, *p* < 0.05). The presence of associated C2 injury suggests that the C2 vertebra was in a more severely injured state due to the occurrence of multiple fractures. Therefore, the presence of this comorbid injury suggests a higher risk of nonunion if the anterior C2 TD fracture is treated conservatively. However, associated C1 injury, C3–7 injury, or thoracolumbar injuries were not statistically different between the union and nonunion groups. In addition, PSVT severity was not statistically different between the union and nonunion groups.

### 4.5. Limitation of Study

The limitations of this study include its retrospective nature. Like other retrospective multicenter studies, we could not completely exclude potential confounders associated with retrospective data collection and selection bias, such as initial treatment methods. In addition, further increase in the number of study subjects was limited by the rarity of this injury, even when pooling data from multiple centers.

## 5. Conclusions

Our results showed that an avulsion fracture ratio > 43%, a fracture displacement > 5 mm, or the presence of associated C2 injury can increase the risk of nonunion for conservatively managed anterior C2 TD fractures. Nonunion, in turn, was associated with inferior clinical outcomes. Therefore, we suggest that surgical treatment could be considered for anterior C2 TD fractures in the presence of these radiological findings.

## Figures and Tables

**Table 1:** Summary of conservatively managed anterior tear drop fractures of C2 (2nd cervical vertebra).

Variables	Union Group (*N* = 26)	Nonunion Group (*N* = 7)	*p*-Value
Age (Years)	51.5 ± 18.3	51.9 ± 19.9	0.961
Sex (Men/Women)	20/6	4/3	0.358
Avulsion Fracture Ratio (%)	29.5 ± 14.6	43.3 ± 11.7	<0.05 *
Fracture Displacement (mm)	3.6 ± 1.5	5.1 ± 0.3	<0.05 *
PSVT Severity			0.988
Grade 1 (Mild)	8 (30.8%)	2 (28.6%)	
Grade 2 (Moderate)	4 (15.4%)	1 (14.3%)	
Grade 3 (Severe)	14 (53.8%)	4 (57.1%)	
Associated C2 Injury	4 (15.4%)	4 (57.1%)	<0.05 **
Associated C1 Injury	1 (3.8%)	1 (12.5%)	0.421
Associated C3–7 or TL Injuries	7 (26.9%)	2 (28.6%)	1.000

PSVT = prevertebral soft tissue thickness; TL = thoracolumbar; * *p*-value calculated by independent *t*-test; ** *p*-value calculated by *chi*-squared test.

**Table 2:** Clinical outcomes using Odom’s criteria in conservatively managed anterior tear drop fractures of C2.

	Union Group (*N* = 26)	Nonunion Group (*N* = 7)	*p*-Value
**Odom’s Criteria**			<0.001
Excellent	16 (61.5%)		
Good	10 (38.5%)	4 (57.1%)	
Fair		3 (42.9%)	
Poor			

*p*-value calculated by *chi*-squared test.

**Table 3:** Clinical outcomes using visual analog scale for neck pain in conservatively managed anterior tear drop fractures of C2.

	Union Group (*N* = 26)	Nonunion Group (*N* = 7)
Initial VAS	4.1 ± 0.7	4.3 ± 0.8
Last Follow-Up VAS	1.2 ± 0.4	3.6 ± 0.5
*p*-Value	<0.001	0.094

VAS = visual analog scale; *p*-value calculated by paired *t*-test.

## Data Availability

All data presented in this study are available on demand from the corresponding author.
